# Exploring the Role of the Connection Length of Screen-Printed Electrodes towards the Hydrogen and Oxygen Evolution Reactions

**DOI:** 10.3390/s23031360

**Published:** 2023-01-25

**Authors:** Phatsawit Wuamprakhon, Alejandro Garcia-Miranda Ferrari, Robert D. Crapnell, Jessica L. Pimlott, Samuel J. Rowley-Neale, Trevor J. Davies, Montree Sawangphruk, Craig E. Banks

**Affiliations:** 1 Faculty of Science and Engineering, Manchester Metropolitan University, Chester Street, Manchester M1 5GD, UK; 2 Centre of Excellence for Energy Storage Technology (CEST), Department of Chemical and Biomolecular Engineering, Vidyasirimedhi Institute of Science and Technology, School of Energy Science and Engineering, Rayong 21210, Thailand; 3 INEOS Electrochemical Solutions, Bankes Lane Office, Bankes Lane, Runcorn, Cheshire WA7 4JE, UK

**Keywords:** hydrogen evolution mechanism, oxygen evolution mechanism, Tafel analysis

## Abstract

Zero-emission hydrogen and oxygen production are critical for the UK to reach net-zero greenhouse gasses by 2050. Electrochemical techniques such as water splitting (electrolysis) coupled with renewables energy can provide a unique approach to achieving zero emissions. Many studies exploring electrocatalysts need to “electrically wire” to their material to measure their performance, which usually involves immobilization upon a solid electrode. We demonstrate that significant differences in the calculated onset potential for both the hydrogen evolution reaction (HER) and oxygen evolution reaction (OER) can be observed when using screen-printed electrodes (SPEs) of differing connection lengths which are immobilized with a range of electrocatalysts. This can lead to false improvements in the reported performance of different electrocatalysts and poor comparisons between the literature. Through the use of electrochemical impedance spectroscopy, uncompensated ohmic resistance can be overcome providing more accurate Tafel analysis.

## 1. Introduction

Facilitating the paradigm shift in the world’s energy economy away from fossil fuel dependency toward less polluting renewable energy sources is vital if the targets set out by the United Nations at the 2021 Climate Change Conference COP26 are to be met [1]. The production of green hydrogen gas through electrolytic water splitting, with the required energy derived from renewable sources such as solar energy, wind energy, tidal energy, biomass energy, etc., is a promising alternative due to its high gravimetric energy density with no direct carbon emissions [2]. To achieve electrolytic water splitting, where both hydrogen and oxygen are produced, a major reaction must occur at both the cathode and anode, namely, the hydrogen evolution reaction and oxygen evolution reaction, respectively. The oxygen evolution reaction (OER) and the hydrogen evolution reaction (HER) are involved in biological and artificial energy conversion schemes [3]. In theory, the splitting of water in this way requires a thermodynamic cell voltage of +1.23 V versus the reversible hydrogen electrode (RHE) [4]. Anything over this is termed an overpotential and indicates thermodynamic inefficiencies within the system.

There are many electrocatalysts that are reported for enhancing the HER and OER [5,6,7]. Numerous papers utilize solid electrodes such as glassy carbon [7], boron-doped diamond [8], nickel foam [5], and screen-printed electrodes to “electrically wire” their electrocatalysts [9,10,11,12,13,14,15,16,17,18,19]. Screen-printed electrodes (SPEs) are manufactured using well-known industrial printers by depositing a combination of layers onto a flat substrate which offers versatility in terms of electrode design, material compatibility, modifications with electrocatalysts, but yet offers highly economical, mass-producible and highly reproducible sensors [20]. In our recent paper [21], we explored, for the first time, changing the connection length of SPEs since their original design has not been explored. We demonstrated that by reducing the connection length, significant improvements were observed in the heterogeneous electrode kinetics and in the performance of electroanalytical sensing platforms. In the field of water splitting, new catalysts are designed, fabricated and then tested via immobilization onto a range of electrode substrates. This “electrically wires” the potentiostat system, allowing the user to test their electrochemical and electrocatalytic performance towards the HER and OER. In the academic literature, many different electrodes are utilized, with SPEs being a main feature due to their low-cost, yet highly reproducible nature; there are many papers that utilize SPEs for hosting their catalysts [9,10,11,15,17,18,19,22]. Note that it is known in the electrochemistry community that the length of the connection cables can add more resistance to the electrochemical system, which ultimately affects the overpotentials. That said, our approach is to explore the effect of the electrode’s length. In this paper, for the first time, the effect of changing the SPE’s connection length for both surface- and bulk-modified water splitting materials towards the HER and OER is studied.

## 2. Materials and Methods

### 2.1. Chemicals and Materials

All chemicals used were of analytical grade and used as received without any further purification. All solutions were prepared with deionized water of resistivity not less than 18.2 MΩ cm (Milli-Q, Millipore, United Kingdom). Molybdenum disulfide (MoS_2_, 99%, 18 mg/L Pristine Flakes) was purchased from Graphene Supermarket (Graphene Laboratories Inc., New York, United States). Potassium ferricyanide, potassium ferrocyanide, potassium chloride, MoSe_2_ (99%), IrO_2_ (99%) and Pt/C (20%) were purchased from Sigma-Aldrich (Merck, Gillingham, United Kingdom). Note that the Pt nanoparticles reported within the commercial Pt/C catalyst are >5 nm in diameter [23]. RuO_2_ (99%) was purchased from Alfa Aesar (Thermo Fisher Scientific, Heysham, United Kingdom). Sulfuric acid (95%) was purchased from Fisher Scientific (Thermo Fisher Scientific, Heysham, United Kingdom).

### 2.2. Electrochemical Measurements

An µ-Autolab Type (III) potentiostat (Autolab, Utrecht, The Netherlands) was used to carry out all electrochemical measurements using a three-electrode configuration. The temperature was experimentally performed at 23 °C. The working electrodes used in this study are screen-printed graphitic macroelectrodes (SPEs) alongside an external Pt wire and Ag|AgCl electrode as the counter and reference electrodes, respectively, unless stated otherwise. All electrochemical measurements (both HER and OER) of the modified SPEs with various lengths were performed in 0.5 M H_2_SO_4_ (pH = 0.18) at room temperature using linear sweep voltammetry (LSV) at 25 mV/s. In the case of the HER, prior to all electrochemical experiments solutions were purged with ultra-pure nitrogen for at least 20 min to remove all oxygen from the system. The potentials of Ag|AgCl were converted to reversible hydrogen electrode (RHE) potential through the following equation:

(1) $$E_{\mathrm{RHE}}=E_{\mathrm{Ag}|\mathrm{AgCl}}+0.059\mathrm{PH}+E_{0}^{\mathrm{Ag}|\mathrm{AgCl}}$$

where E^0^_Ag|AgCl_ is 0.1976 V. Electrochemical impedance spectroscopy (EIS) (50,000–1 Hz, amplitude = 10 mV) was performed in ferri/ferrocyanide (1 mM, 0.1 M KCl) at + 0.23 V vs. Ag|AgCl, taking the resistance value from the fit obtained through circuit fitting, as described previously [21].

### 2.3. Screen-Printed Electrode Fabrication

**Figure 1:**
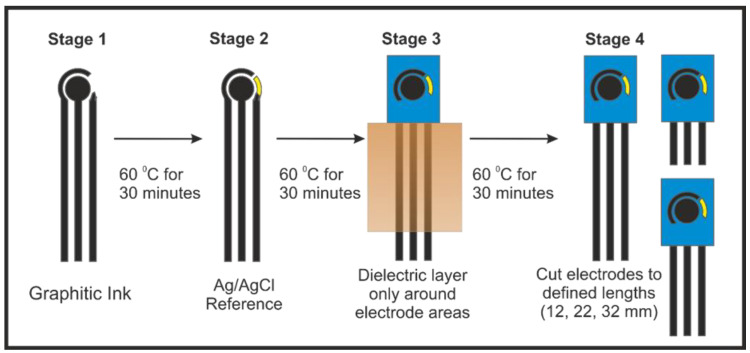
Schematic representation of the production of the screen-printed electrodes with various connection lengths used throughout this work. The bulk-modified SPEs had an additional working electrode layer printed, using the same graphitic ink modified with 10% (*w*/*w*) catalyst before being dried at 60 °C for 30 min. Reproduced with permission from ref. [21]. Copyright 2021 American Chemical Society (ACS).

SPE manufacturing was achieved following our previous reports [21], where electrodes were produced with varying connection lengths and were then electrically wired via an edge connector, which then connected to the potentiostat. In this paper, we compare the use of 32, 27, 22, 17 and 12 mm long × 7 mm wide connections for our SPE systems. Figure 1 shows an overview of how the production of the screen-printed electrodes with various connection lengths were used throughout this work.

**Figure 2:**
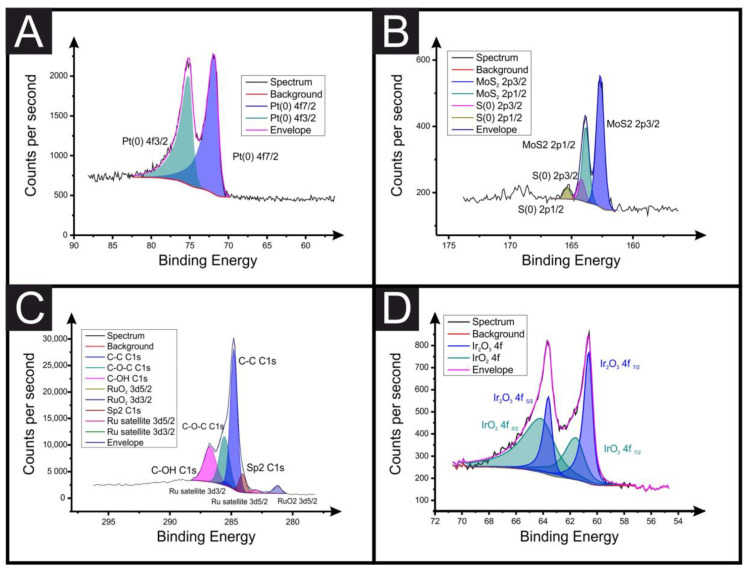
XPS data corresponding to the working electrode surface of the bulk-modified SPEs, highlighting the incorporation of the Pt/C, MoS_2_, RuO_2_ and IrO_2_ (**A**–**D**, respectively) electrocatalysts.

The SPEs are made by a three-electrode configuration comprised of a 3.1 mm graphite working electrode, a graphite counter and an Ag|AgCl pseudo-reference electrode; however, for all electrochemical experiments in this manuscript, external counter and reference electrodes were used (Ni wire and Ag|AgCl, respectively), unless otherwise stated. The in-house-manufactured SPE platforms were printed using a microDEK 1760RS screen-printing machine (DEK Printing Machines Ltd., Weymouth, United Kingdom). The first graphitic layer was made with a carbon graphite ink formulation (Product Code: C2000802P2; Gwent Electronic Materials Ltd, Pontypool, United Kingdom), which was deposited onto a polyester (Autostat, 250 micron thickness) substrate. Fan-assisted curation of the layer at 60 °C for 30 minutes followed. Finally, to define the electrodes and protect exposed circuits, a dielectric paste (Product Code: D2070423D5; Gwent Electronic Materials Ltd, Pontypool, United Kingdom) was printed onto the polyester substrate to cover the connections. A polyimide tape was used to define/cover a fixed section of the screen so only the working end of the SPE system was printed with dielectric. The SPEs were then cut to the chosen lengths. Another curation step at 60 °C for 30 minutes followed. The reproducibility of the batch of screen-printed electrode were found to correspond to less than 4.5% RSD towards the redox probe, [Ru(NH_3_)]^2+/3⁺^/0.1 M KCl. Edge connectors were used to connect the potentiostat connections to the SPEs [24]. The surface-modified (drop-casting) method was used to load all electrocatalysts on the SPEs (with 1542 ng/cm^2^ mass loading of catalyst), where an aliquot of ink solution was added onto the SPEs’ surfaces using a manual micropipette. The modified SPEs were dried at ambient temperature. These are termed as SPE_surface_. In the case of powder catalysts, powder was dispersed in DI water and further sonicated for 10 minutes to make the ink solution. For the bulk-modified catalyst SPEs, an extra ink deposition followed, made by a 10% catalyst loading in weight, which was then printed onto the defined working electrode area only. These are termed as SPE_bulk_. In the case of the bare SPEs, the resistances measured through EIS for the different-length SPEs were found to be 32 mm = 2.16 ± 0.06 kΩ; 27 mm = 1.79 ± 0.05 kΩ; 22 mm = 1.50 ± 0.03 kΩ; 17 mm = 1.32 ± 0.04 kΩ; and 12 mm = 0.090 ± 0.04 kΩ. These were measured through EIS (50,000–1 Hz, amplitude = 10 mV) in ferri/ferrocyanide (1 mM, 0.1 M KCl) at +0.23 V vs. Ag|AgCl, taking the resistance value from the fit obtained through circuit fitting, as described previously [21]. All voltammetric experiments were performed at 22 °C. Confirmation of the presence of the catalysts was obtained through XPS measurements, as shown in Figure 2. In the case of IrO_2_, the XPS spectra of the Ir*4f* core levels had Ir_2_O_3_ and IrO_2_ compositions. Peak fitting was performed using Shirley background subtraction and Gaussian–Lorentzian functions. The two peak positions of the x-ray photo-electron spectra are strong indications of the presence of Ir_2_O_3_ *4f_7/2_* ( 26.34 at. %) and Ir_2_O_3_ *4f_5/2_* (15.03 at. %) spin orbital components. In addition, IrO_2_ *4f_7/2_* ( 19.08 at. %) and IrO_2_ *4f_5/2_* (39.55 at. %) doublets are observed, which equates to a ratio of 41.37: 58.63 for Ir_2_O_3_: IrO_2_; such analysis confirms that the material is composed of IrO_2_.

## 3. Results and Discussion

**Figure 3:**
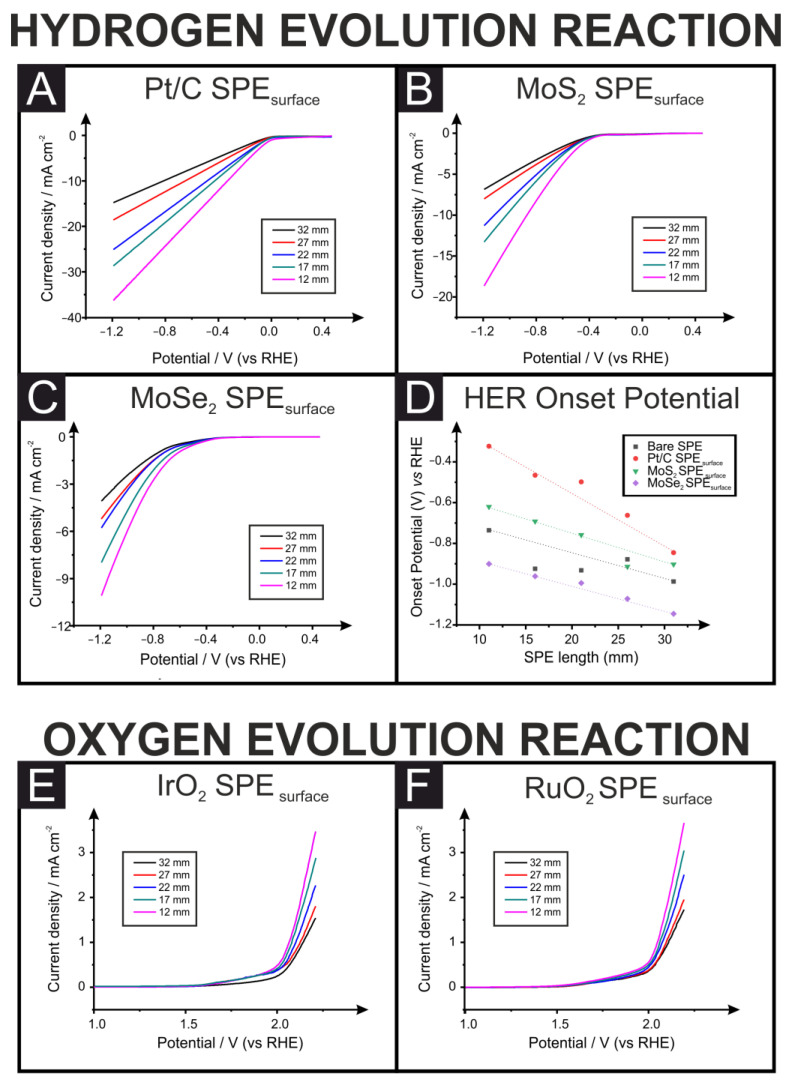
LSV for the different drop-casted SPEs (SPE_surface_) with different connection lengths towards the HER (**A**) Pt/C, (**B**) MoS_2_, and (**C**) MoSe_2_; (**D**) a comparison of the measured overpotential for each catalyst at different SPE connection lengths. Additionally, LSVs are shown for the drop-casted SPEs with different connection lengths towards the OER (IrO_2_ (**E**) and RuO_2_ (**F**). LSVs were recorded in 0.5 M H_2_SO_4_ using a three-electrode set-up consisting of an SPE working electrode, a Pt wire counter electrode and a Ag|AgCl reference electrode.

Figure 3 presents the linear sweep voltammograms (LSV) for the HER (A–D) and OER (E–F) in 0.5 M H_2_SO_4_, obtained using different electrocatalysts that are widely utilized and reported throughout the literature, drop-cast onto the surface of SPEs of varying connection lengths (12, 17, 22, 27 and 32 mm) at a specific mass loading of 1542 ng cm^−1^, as different mass loadings would alter the total number of active sites. The HER/OER electrochemical reactions at the electrodes are greatly impacted by the catalysts’ electrical conductivity, as well as the triple phase boundaries [25,26,27,28], but we have chosen to limit our studies to one specific mass loading to show the effect of changing the SPE’s length on the HER/OER.

In all cases, the only difference between systems of the same catalyst is the connection length of the SPE. The resistances measured through EIS for the different-length SPEs were: 32 mm = 2.16 ± 0.06 kΩ; 27 mm = 1.79 ± 0.05 kΩ; 22 mm = 1.50 ± 0.03 kΩ; 17 mm = 1.32 ± 0.03 kΩ; and 12 mm = 0.090 ± 0.03 kΩ. It is clear that, for all cases, a trend is observed where as the connection length of the surface-modified SPEs is reduced, the system’s performance toward the reaction under study is enhanced. For both the HER and OER, the onset overpotential is determined as the potential at which the measured current deviates from the background current by the value of 25 µA cm^−2^ [29,30]. Table 1 provides a summary comparison of the effect changing the connection length of the SPE has on the measured overpotential towards the HER.

It is clear that SPE connection length has a profound effect on this value for each catalyst, where a change from 32 mm to 12 mm for the SPE system can improve the HER overpotential (ca. 62, 31 and 21% when using Pt/C, MoS_2_ and MoSe_2_ surface-modified SPEs, respectively). Note that the position of the HER and OER peaks in our work are in agreement with independent reports [31]. Therefore, two individuals researching identical catalytic systems would produce entirely different results based solely upon the connection length of the SPEs they use. As such, any future work utilising these substrates for energy applications should include a full characterization of the resistance of each electrode used through EIS or, as a minimum, a simple multi-meter measurement.

In addition to simple drop-casting of the chosen catalyst onto the working electrode’s surface, SPEs allow the researcher to modify their bulk (denoted as SPE_bulk_) such that the catalyst is “anchored” into the graphitic ink used to produce the SPE [32,33,34]. These SPEs were produced in the same manner as a previous study [21] to allow for simple modification of the connection length (Figure 1).

**Figure 4:**
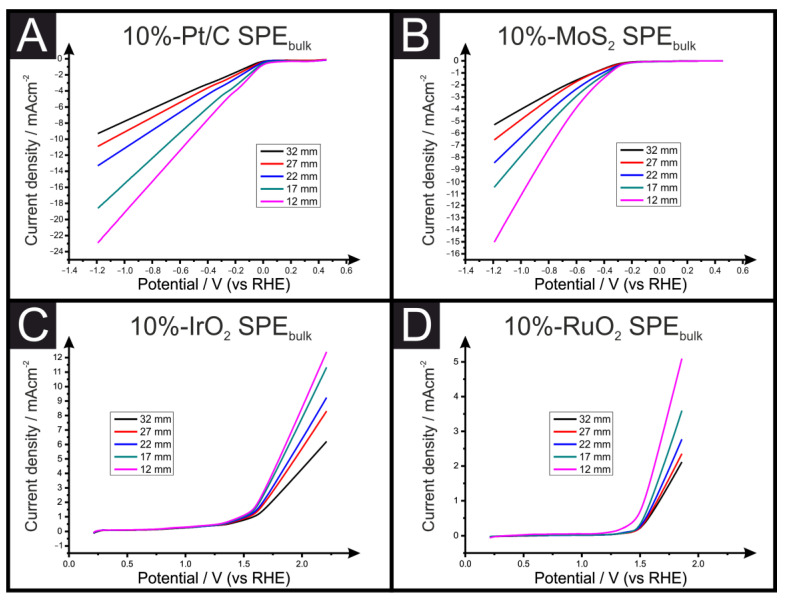
LSV for the different bulk-modified (10%) SPEs (SPE_bulk_) with different connection lengths towards the HER (Pt/C (**A** ) and MoS_2_ (**B**) alongside LSVs for the bulk-modified (10%) SPEs with different connection lengths towards the OER (IrO_2_ (**C**) and RuO_2_ (**D**). LSVs were recorded in 0.5 M H_2_SO_4_ using a three-electrode set-up consisting of an SPE working electrode, a Pt wire counter electrode and a Ag|AgCl reference electrode.

Figure 4 shows the LSVs obtained for Pt/C and MoS_2_ bulk-modified SPEs toward the HER, alongside IrO_2_ and RuO_2_ bulk-modified SPEs toward the OER. In all cases, again, the SPE connection length produced a clear effect on the obtained electrochemical performance, including the measured overpotential. For example, in the case of the 10% MoS_2_ bulk-modified SPE, the measured overpotential toward the HER improved from a value of −0.37 to −0.30 V (vs. RHE). Table 2 compares the overpotentials measured for the OER catalysts in both the SPE_surface_ and SPE_bulk_ forms with connection lengths varying from 32 to 12 mm. The SPEs containing 10% RuO_2_ had improved onset potentials towards the OER from 1.57 V (vs. RHE) for the 32 mm connection length to 1.47 V (vs. RHE) for the 12 mm connection length. These significant changes of 100 mV can help explain to researchers why their electrocatalyst is better than others. The reason for these observations is that reducing the electrode connection length results in a reduction in the ohmic resistance of the SPEs and the electron transfer pathway decreases. We have recently explored and reported the use of multimeters and EIS for the calculation of ohmic drops (R_u_) and the intrinsic resistance of the electrochemical cell (R_c_), and we suggest that this experimental value be measured and reported to allow for literature comparisons [21].

We utilized DigiElch to explore the theoretical effect of ohmic drop, $R_{u}$, on the HER, which is shown in Figure 3, following Butler–Volmer kinetics/theory and through modelling Equation (1) and (2). Note that we have removed the peak current that is observed from the simulation software but is not seen experimentally, where significant bubbling would be observed and the mass transport characteristics would change. It is evident, as shown in Figure 3, that the effect of ohmic drop is significant and will affect the obtained polarization curve and electrochemical data, resulting in different (unreliable) Tafel values. Returning to the case of the HER, in acidic solutions on platinum, the mechanism proceeds via the Volmer reaction followed by either the Heyrovsky or Tafel reactions with the corresponding Tafel values:

$$\mathrm{Volmer}:\;H^{+}\left(aq\right)+e^{-}\left(m\right)\to H^{\cdot }\left(ads\right);$$

(2) $$\mathrm{Tafel}=2.3\mathrm{RT}/\mathrm{aF}\sim 120\mathrm{mV}{\mathrm{dec}}^{-1}$$

followed by either:

$$\mathrm{Heyrovsky}:\;H^{+}\left(aq\right)+H^{\cdot }\left(ads\right)+e^{-}\left(m\right)\to H_{2}\left(g\right)$$

(3) $$\mathrm{Tafel}=2.3\mathrm{RT}/(1+a)F\sim 40\mathrm{mV}{\mathrm{dec}}^{-1}$$

$$\mathrm{Tafel}:\;H\left(\mathrm{ads}\right)+H\left(\mathrm{ads}\right)\to H_{2}\left(g\right)$$

(4) $$\mathrm{Tafel}=2.3\mathrm{RT}/2F\sim 30\mathrm{mV}{\mathrm{dec}}^{-1}$$

Through the use of a Tafel plot, the rate determining step can be deduced. Thus, it is clear that the Tafel plot is a crucial equation in helping to identify electrochemical mechanisms. Relating this to the data presented in Figure 3 and Figure 4, it is evident that the resistance of the SPE dramatically affects the HER (and OER) such that more reliable electrochemical data will be obtained using an electrode with the lowest resistance and, in our case, the shortest SPE. If there is uncompensated ohmic resistance (i.e., solution resistance, contact resistance), it will distort the observed polarization curve, and the Tafel slope will be effected (i.e., it will be affected by resistance as well as by kinetics).

**Figure 5:**
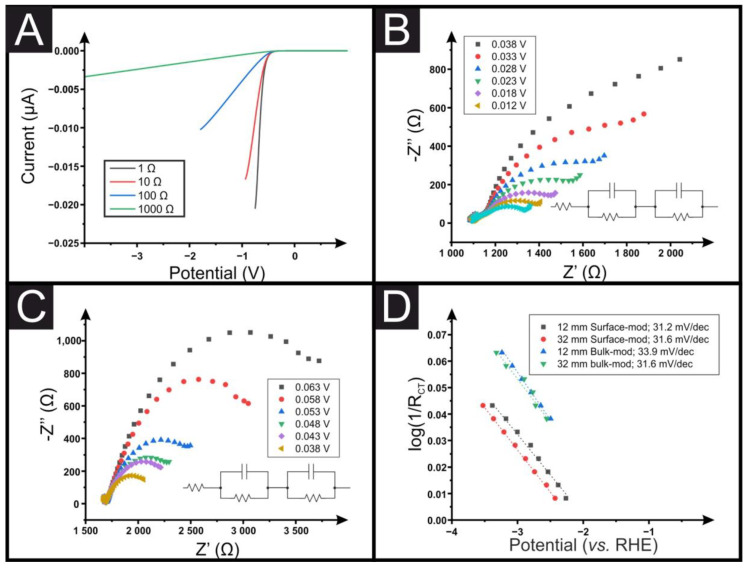
(**A**) Plot of the simulated LSVs for the HER with different resistance electrodes (1, 10, 100 and 1000 Ω). Simulation parameters: scan rate = 100 mV s^−1^; temperature = 298.2 K; electrode area = 0.07 cm^2^; heterogeneous rate constant ($k^{0}$) = 1 × 10^−7^ cm s^−1^ [37]; concentration = 0.5 mol L^−1^; diffusion coefficient of H^+^ = 8.8 × 10^−5^ cm^2^ s^−1^ [38]. (**B**) EIS spectra for the HER using the Pt/C surface-modified 12 mm SPE at different applied potentials. Performed in H_2_SO_4_ (0.5 M). (**C**) EIS spectra for the HER using the Pt/C bulk-modified 32 mm SPE at different applied potentials. Performed in H_2_SO_4_ (0.5 M). (**D**) Plot of the log(1/R_CT_) vs. applied potential for the Pt/C surface-modified and bulk-modified 32 and 12 mm SPEs.

The effect that a change in the resistance will have on the obtained LSV is presented in Figure 5A through simulation data. This figure shows the dramatic change that can occur in obtained data from exponential increases in the system resistance. If the solution resistance is known/measured, it is possible to correct the polarization curve—this might produce a more reliable Tafel plot, but other parameters might be affecting the system concurrently. Essentially, a polarization curve is needed that is only affected by kinetics, which can be difficult to achieve with coated electrodes—impedance suggests that some of the ohmic resistance of the cell is not accounted for by the high-frequency resistance. Therefore, even if the polarization curves were corrected for solution resistance (using the high-frequency resistance from the impedance measurements), they would still be distorted. As we have seen above, the slope from a standard Tafel plot can be significantly altered depending on the resistance of the electrode substrate used to electrochemically wire the catalyst under observation. An alternative way to generate the value for the Tafel slope is through EIS at suitable potentials, reflecting the charge transfer ability of the system [35,36]. In this approach, the usual Tafel polarization curve should be obtained, that is, monitoring the current as a function of potential to show where the HER occurs, e.g., see Figure 2A. EIS should then be applied at suitable potentials. Figure 5B shows the EIS spectra of theoretical versus real impedance (Z″ and Z′) and corresponding analysis obtained for the surface-modified Pt/C catalyst towards the HER for the system using an SPE with a 12 mm connection length, and Figure 5C shows the results for the bulk-modified SPE with a 32 mm connection length. The EIS data for the surface-modified systems show two semi-circular plots, one consistently overlapping at all potentials at a set connection length corresponding to the SPE surface and one becoming significantly reduced at more negative potentials corresponding to the Pt/C at which the HER is occurring. Through fitting with an appropriate RC circuit to match the changing catalyst’s semi-circle, the charge-transfer resistance (R_CT_) can be obtained. This can be utilized in a plot of log(1/R_CT_) vs. the potential, which is equal to the inverse of the Tafel slope and is independent of the SPE’s resistance and other resistances (solution resistance, etc.). It is interesting to note that when using a bulk-modified SPE, as in Figure 3C, there is only a single semi-circle due to the layers being combined. Even so, the same fitting rules can be applied to obtain suitable inverse Tafel slopes. Figure 5D shows this for the surface-modified Pt/C catalyst using SPEs of 32 and 12 mm connection lengths, where gradients of 31.6 and 31.2 mV dec^−1^ are obtained, and bulk-modified Pt/C catalyst using an SPE of 32 and 12 mm connection lengths, producing gradients of 31.6 and 33.9 mV dec^−1^, respectively, all within experimental error. This confirms that the Tafel step in Equation (3) is the rate determining step in both cases, as expected. This approach shows how using EIS can produce unambiguous results in terms of catalysts toward the HER in addition to the OER, which has been described previously. We would recommend this methodology moving forward to improve the consistency in this field and allow for easier and more transparent catalyst comparison, independent of the electrode substrate used.

## 4. Conclusions

In summary, we have shown for the first time that the SPE connection length is a critical parameter and has a profound effect on the overpotential of each electrocatalyst toward both the HER and OER when used as the basis to electrically wire the catalyst to the electrochemical system. It is important to note that the majority of the academic literature relies on the measurement and reporting on overpotentials to justify their material as a “good” electrocatalyst, and as such, when SPEs are used, the ohmic resistance should be considered and reported. Additionally, we report the use of EIS to calculate unambiguous results in regard to the Tafel slope, allowing simpler and more transparent comparisons between catalysts, independent of the electrode substrate used by the experimentalist. Future work that reports Tafel slopes should use the EIS method to remove ambiguity, as well as report the ohmic resistance for all electrodes that are used to “electrically wire” their electrocatalysts.

## Figures and Tables

**Table 1:** Comparison of the relative overpotential values, expressed as percentages, for the HER catalysts Pt/C, MoS_2_ and MoSe_2_ when surface-modified onto SPEs of different connection lengths. Measured in 0.5 M H_2_SO_4_ with potentials reported vs. the reversible hydrogen electrode (RHE).

Connection Length (mm).	Pt/C-SPE_surface_ Overpotential %	MoS_2_-SPE_surface_ Overpotential %	MoSe_2_-SPE_surface_ Overpotential %
32	0.0	0.0	0.0
27	−21.7	1.2	−6.4
22	−41.0	−16.0	−13.2
17	−44.9	−23.4	−16.1
12	−61.7	−31.3	−21.4

**Table 2:** Summary of the onset potentials for the RuO_2_ and IrO_2_ catalysts, both as surface-modified and bulk-modified, on SPEs of different connection lengths. Measured in 0.5 M H_2_SO_4_ with potentials reported vs. the reversible hydrogen electrode (RHE).

Connection Length (mm).	RuO_2_-SPE_surface_ (V)	RuO_2_-SPE_bulk_ (V)	IrO_2_-SPE_surface_ (V)	IrO_2_-SPE_bulk_ (V)
32	1.58	1.52	1.57	1.37
27	1.57	1.52	1.56	1.35
22	1.56	1.45	1.55	1.33
17	1.51	1.44	1.53	1.31
12	1.49	1.39	1.47	1.29
